# The DNA Minor Groove Binders Trabectedin and Lurbinectedin Are Potent Antitumor Agents in Human Intrahepatic Cholangiocarcinoma

**DOI:** 10.3390/ijms26189085

**Published:** 2025-09-18

**Authors:** Erwin Gäbele, Isabella Gigante, Mirella Pastore, Antonio Cigliano, Grazia Galleri, Thea Bauer, Elena Pizzuto, Serena Mancarella, Martina Müller, Fabio Marra, Heiko Siegmund, Gianluigi Giannelli, Matthias Evert, Chiara Raggi, Diego F. Calvisi, Sara M. Steinmann

**Affiliations:** 1 Department of Internal Medicine I, University Hospital Regensburg, 93053 Regensburg, Germany; gaebele@internisten-regensburg.de (E.G.); martina.mueller-schilling@ukr.de (M.M.); 2 Internisten-Regensburg.de, Internal Medicine Group Practice, 93053 Regensburg, Germany; 3 National Institute of Gastroenterology, IRCCS “Saverio de Bellis”, 70013 Castellana Grotte, Italy; isabella.gigante@irccsdebellis.it (I.G.); elena.pizzuto@irccsdebellis.it (E.P.); serena.mancarella@irccsdebellis.it (S.M.); gianluigi.giannelli@irccsdebellis.it (G.G.); 4 Department of Experimental and Clinical Medicine, University of Florence, 50121 Florence, Italy; mirella.pastore@unifi.it (M.P.); fabio.marra@unifi.it (F.M.); chiara.raggi@unifi.it (C.R.); 5 Department of Medicine, Surgery, and Pharmacy, University of Sassari, 07100 Sassari, Italy; acigliano@uniss.it; 6 Department of Biomedical Sciences, University of Sassari, 07100 Sassari, Italy; galleri@uniss.it; 7 Institute of Pathology, University of Regensburg, 93053 Regensburg, Germany; thea.bauer@stud.uni-regensburg.de (T.B.); heiko.siegmund@ukr.de (H.S.); matthias.evert@klinik.uni-regensburg.de (M.E.); diego.calvisi@klinik.uni-regensburg.de (D.F.C.)

**Keywords:** intrahepatic cholangiocarcinoma, trabectedin, lurbinectedin, innovative therapies, DNA damage response, patient-derived tumor organoids (PDOs), cancer-associated fibroblasts (CAFs), apoptosis

## Abstract

Intrahepatic cholangiocarcinoma (iCCA) is the second most common primary liver tumor. Due to its aggressive nature and resistance to conventional treatments, there is a pressing need to develop novel and more effective therapies for this deadly malignancy. Here, we explored the therapeutic potential of the DNA minor groove binders trabectedin (TRB) and lurbinectedin (LUR) for the treatment of iCCA using cell lines, spheroids, cancer-associated fibroblasts (CAFs), patient-derived tumor organoids (PDOs), and the chicken chorioallantoic membrane (CAM) in vivo model. TRB and, more substantially, LUR, significantly inhibited cell growth in iCCA cell lines, spheroids, CAFs, and PDOs at very low nanomolar concentrations. Specifically, the two drugs significantly reduced proliferation, triggered apoptosis, and caused DNA damage in iCCA cells. At the metabolic level, TRB and LUR decreased mitochondrial respiration and glycolysis. At the molecular level, the two compounds effectively downregulated the mammalian target of rapamycin complex 1 (mTORC1) and Hippo/YAP pathways and suppressed the expression of yes-associated protein 1 (YAP1), cellular myelocytomatosis oncogene (c-Myc), E2F transcription factor 1 (E2F1), Bromodomain-containing protein 4 (BRD4), TEA domain transcription factor 4 (TEAD4), and cluster of differentiation 7 (CD7) proto-oncogenes. Furthermore, LUR significantly restrained the in vivo growth of iCCA cells in the CAM model. Our data indicate that TRB and LUR possess strong anti-proliferative and pro-apoptotic activities and could represent promising therapeutic agents for the treatment of iCCA.

## 1. Introduction

Intrahepatic cholangiocarcinoma (iCCA) is the second most common primary liver cancer, arising from the epithelial cells of the intrahepatic bile ducts and accounting for approximately 15% to 20% of all liver malignancies [1,2]. In the past 40 years, iCCA diagnoses in the United States have increased by 128%. Despite this ongoing rise in morbidity and mortality, the disease remains challenging to detect and manage due to the lack of specific symptoms, its intrinsic aggressiveness, and the limited therapeutic options available [3].

The primary risk factors associated with the development of iCCA include primary sclerosing cholangitis, fibro-polycystic liver disease, chronic infections with hepatitis B and C viruses, hepatobiliary flukes, hepatolithiasis, and metabolic dysfunction-associated steatotic liver disease (MASLD). However, in many cases, iCCA occurs without any identifiable risk factors [4]. Surgical resection is considered the only potentially curative treatment for this condition. Unfortunately, only 20% to 30% of patients are eligible for surgery because symptoms often do not appear in the early stages of the disease. Consequently, most patients are diagnosed at an advanced stage, where the 5-year survival rate is usually between 5% and 10% [5]. For patients with unresectable tumors or those with distant metastasis, the primary treatment consists of a combination of cisplatin and gemcitabine chemotherapy. Second-line treatment options include 5-Fluorouracil (5-FU) and oxaliplatin or irinotecan [6,7]. Even with these treatments, the high heterogeneity of iCCA often results in poor outcomes, with an objective response rate of approximately 5% and a median overall survival of just 6.2 months [8].

There is a growing trend toward combining systemic therapy, locoregional therapies, and molecular approaches [9]. Chemo-immunotherapy is becoming the first-line standard of care for unresectable iCCA, supported by the results of phase III TOPAZ-1 and KEYNOTE-966 trials [10,11]. Additionally, the U.S. Food and Drug Administration (FDA) and the European Medicines Agency (EMA) have recently approved personalized therapies targeting alterations in fibroblast growth factor receptor (FGFR) and isocitrate dehydrogenase (IDH). These alterations occur in approximately 15% to 20% of iCCA cases [12,13,14,15]. Unfortunately, the benefits of these therapeutic approaches remain unsatisfactory, either due to their applicability to limited patient cohorts or because the emergence of resistance mechanisms undermines long-term survival.

Recent studies have concentrated on synthesizing new compounds or screening bioactive components from natural products to create innovative treatments for unresectable tumors. In our pursuit of new approaches for treating iCCA, we began investigating the anticancer drugs trabectedin (TRB; Yondelis, ET-743) and lurbinectedin (LUR; Zepzelca) [16,17]. TRB is a natural compound derived from the Caribbean Sea squirt, *Ecteinascidia turbinata*. It belongs to the marine-based tetrahydroisoquinoline family of antitumor agents and is now produced entirely through synthetic methods. This drug acts as a powerful alkylator by interacting with the minor groove of DNA, leading to cytotoxic effects and impacting DNA repair pathways [18]. LUR is a synthetic alkaloid analog of TRB. It has a more favorable toxicity profile and improved pharmacokinetics compared to TRB [19]. At the molecular level, both drugs bind to the minor groove of DNA. This binding stops transcription and causes a delayed transition through the S phase of the cell cycle. Eventually, this leads to cell cycle arrest in the G2-M phase and cell death through apoptosis [20,21]. This specific activity in the G2 phase is unique, especially compared to other alkylating agents that usually act during the S phase. Furthermore, LUR specifically targets GC-rich sequences within the promoters of certain genes, leading to the degradation of elongating RNA polymerase II through the ubiquitin-proteasome machinery. As a result, single-strand and double-strand DNA breaks arise, contributing to cell death [20,22,23]. Recent studies have shown that these compounds form adducts by binding to guanine in the minor groove of DNA, resulting in a bend toward the major groove [24]. The generated adducts help displace oncogenic transcription factors from their target promoters, negatively affecting oncogenic pathways [25,26].

TRB is used as a second-line treatment in the clinical setting for advanced soft tissue sarcoma, particularly liposarcoma and leiomyosarcoma, as well as for relapsed platinum-sensitive ovarian cancer when combined with pegylated liposomal doxorubicin [27,28,29,30]. LUR is indicated for the treatment of adults with metastatic small cell lung cancer (SCLC) who experience disease progression on or after platinum-based chemotherapy. Clinical trials have shown a response rate of 37% to 67%, with myelosuppression being the main side effect. In addition to myelosuppression, both drugs share common primary toxicities, including a transient increase in hepatic transaminases, nausea, vomiting, and fatigue [17,31]. Furthermore, both TRB and LUR are primarily metabolized by Cytochrome P450 3A4 (CYP3A4), meaning that inducers or inhibitors of this enzyme could impact their clearance rates [32].

Notably, both TRB and LUR not only affect cancer cells but also have a significant impact on the tumor microenvironment (TME). This includes immune cells such as monocytes, macrophages, and tumor-associated macrophages (TAMs), as well as the blood vessels within the tumor. These compounds work by blocking various inflammatory mediators, reducing monocyte adhesion, and influencing the expression of genes involved in the organization of the actin cytoskeleton. Additionally, they hinder the production of angiogenic factors that are essential for tumor growth and progression [33]. This ability to modulate the immune response could be beneficial when combined with other immunostimulatory approaches, such as checkpoint blockade immunotherapies [34]. Due to their effectiveness in targeting both cancer cells and the TME, multiple clinical trials have been conducted to evaluate the efficacy and safety of these drugs, either alone or in combination with other treatments. These trials involve various solid malignancies (NCT02454972, NCT05126433, NCT02210364, NCT00147212, NCT00050427).

In this study, we conducted a comprehensive investigation into the effectiveness and mechanisms of action of TRB and LUR in various in vitro models of iCCA. Our findings indicate that these two drugs show promise as therapeutic candidates for treating this aggressive type of cancer.

## 2. Results

### 2.1. Trabectedin and Lurbinectedin Restrain Cell Growth and Trigger DNA Damage in iCCA Cell Lines

**Figure 1:**
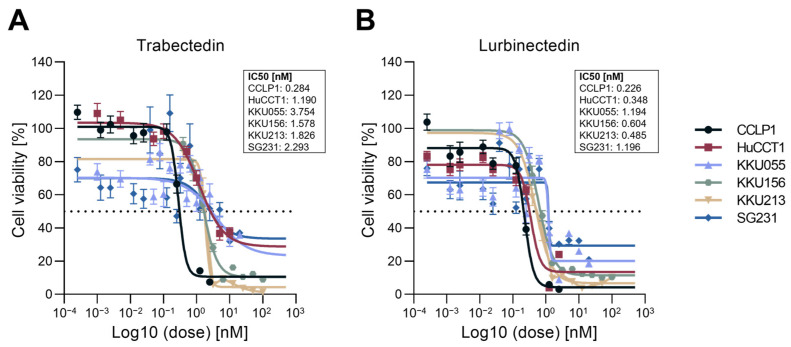
Effect of trabectedin and lurbinectedin on the viability of intrahepatic cholangiocarcinoma cell lines. Cell viability of six human intrahepatic cholangiocarinoma (iCCA) cell lines (CCLP1, HUCCT1, KKU055, KKU156, KKU213, and SG231) exposed to 0–100 nM trabectedin (**A**) and lurbinectedin (**B**) for 48 h was assessed by MTT assay. Data from three independent experiments, each with eight replicates, are represented as the mean percentage of DMSO-treated cells ± standard deviation. A summary of the IC_50_ of the drug in the six cell lines is depicted next to the graph. Dashed horizontal lines indicate 50% cell viability.

To determine the cytotoxic potential of TRB and LUR in iCCA, the two DNA minor groove binders were administered to six human iCCA cell lines (HUCCT1, CCLP1, KKU213, KKU156, KKU055, and SG231). The cell lines were treated with concentrations between 0 and 100 nM of TRB and LUR, and colorimetric MTT assays were performed 48 h after drug administration. Both drugs profoundly reduced tumor cell viability in the six cell lines tested in a dose-dependent manner (Figure 1A,B). Notably, a significant anti-growth effect of the two compounds was observed at low nanomolar concentrations in all tested cell lines, with LUR demonstrating the lowest IC_50_ values across all lines.

**Figure 2:**
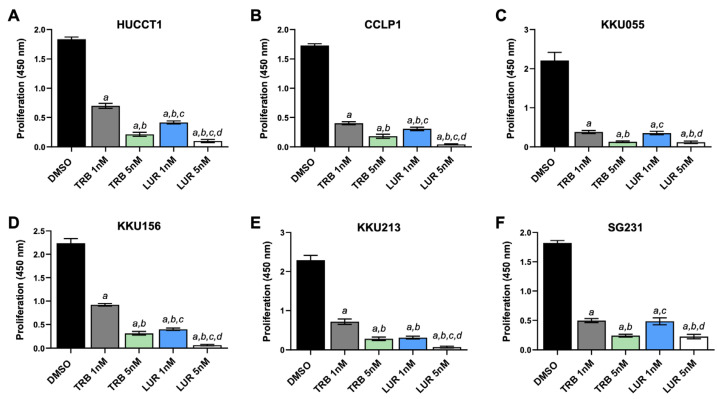
Effects of trabectedin and lurbinectedin on the proliferation of intrahepatic cholangiocarcinoma cell line monolayer cultures indicated by BrdU incorporation. BrdU incorporation assay was conducted on HUCCT1 (**A**), CCLP1 (**B**), KKU055 (**C**), KKU156 (**D**), KKU213 (**E**), and SG231 (**F**) cells treated for 48 h with trabectedin (TRB) and lurbinectedin (LUR) at 1 nM and 5 nM concentrations. Cells treated with solvent (DMSO) served as controls. Results are expressed as mean ± standard deviation of three independent experiments in triplicate. For statistical analysis, Tukey’s multiple comparisons test was performed; at least *p* < 0.01; a, vs. DMSO; b, vs. 1 nM TRB; c, vs. 5 nM TRB; d, vs. 1 nM LUR.

**Figure 3:**
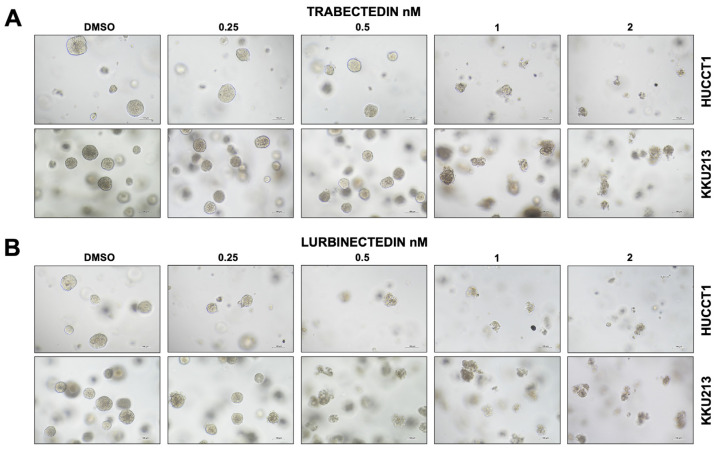
Trabectedin and lurbinectedin restrain the growth of HUCCT1 and KKU213 intrahepatic cholangiocarcinoma cells grown as 3D cultures (spheroids). HUCCT1- and KKU213-derived spheroids were treated with DMSO (vehicle) and increasing concentrations of trabectedin (TRB) and lurbinectedin (LUR; 0.25–2 nM; *n* = 3 wells per condition). Note the decrease in size and number of spheroids following the treatment with TRB (**A**) and LUR (**B**), as assessed by the optical microscope. Original magnification: 10×; scale bar: 100 µm.

To further elucidate the mechanisms of action of the two drugs in iCCA cells, the six cell lines were subjected to proliferation and apoptosis assays. As concerns cell proliferation, incubation with TRB and LUR at increasing concentrations (1 and 5 nM) significantly reduced bromodeoxyuridine (BrdU) incorporation in the six cell lines, often with a more pronounced constraining effect induced by LUR, in a dose-dependent manner (Figure 2A–F). Regarding apoptosis, TRB and LUR induced robust apoptotic cell death in the six cell lines, with a slightly higher cell death in most LUR-treated cells (Supplementary Figure S1A–F). Similarly, TRB and LUR were effective in reducing the growth of tumor spheres (SPH) generated from HUCCT1 and KKU213 cell lines in a dose-dependent manner. In particular, HUCCT1 and KKU213 spheroids showed a dramatic reduction in viability even with the lowest concentration of LUR used, whereas increasing doses of TRB induced a dose–response curve in terms of cell viability in both iCCA cell spheroids (Figure 3A,B, Supplementary Figure S2A–D). Equivalent results were obtained in spheroids from CCLP1 and SG231 cells. As we previously demonstrated, spheroids exhibit a significant increase in stemness-related features compared to monolayer cultures of the parental cells [35,36].

**Figure 4:**
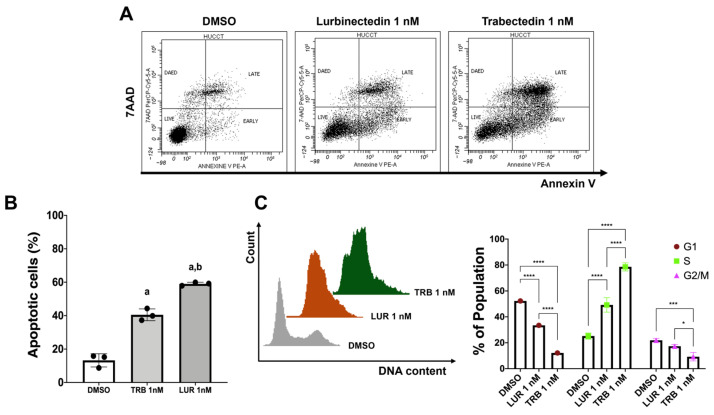
Effect of lurbinectedin and trabectedin on the apoptosis and cell cycle of intrahepatic cholangiocarcinoma cell lines, as assessed by flow cytometry analysis. (**A**) Dot plot graph of the apoptotic analysis representative of three independent experiments. Apoptotic analysis with Annexin V-PE and 7-AAD staining of HUCCT1 intrahepatic cholangiocarcinoma (iCCA) cells treated with trabectedin (TRB) and lurbinectedin (LUR) at 1 nM concentration for 24 h. (**B**) Data as the percentage of total apoptotic cells, are presented as mean ± standard deviation (SD) of three independent experiments, and the significance level of ANOVA is reported (*p* < 0.001) according to Tukey’s multiple comparisons test. Lowercase letters are used to denote statistical significance (a, vs. Vehicle; b, vs. 1 nM TRB). (**C**) Left: Representative images of cell cycle distribution of iCCA cells that were cultured in complete medium and treated with vehicle or 1 nM LUR or 1 nM TRB for 24 h. Right: Quantification of the cell cycle phases from three independent experiments. Data are presented as mean ± SD and were analyzed using Tukey’s multiple comparisons test (* *p* < 0.05, *** *p* < 0.001, **** *p* < 0.0001).

To further substantiate our findings, randomly selected HUCCT1, KKU156, and KKU213 cell lines were treated with TRB and LUR at 1 nM concentration, and flow cytometry analysis was performed 24 h after starting drug exposure. First, we examined the apoptosis induced by the two compounds on the three cell lines, which was measured using Annexin V-phycoerythrin (PE) and 7-aminoactinomycin D (7-AAD). Treatment with the two drugs resulted in a substantial increase in cell apoptosis compared to the vehicle (Figure 4A,B, and Supplementary Figures S3A,B and S4A,B). Next, to further investigate the inhibition of cell proliferation, cell cycle analysis was performed to evaluate the effects of TRB and LUR on cell cycle phase distribution (Figure 4C and Supplementary Figures S3C and S4C) in HUCCT1, KKU156, and KKU213 cell lines. In these cells, treatment with 1 nM TRB and LUR compared to vehicle affected predominantly the S/G2 transition of the cell cycle with an evident accumulation in the S phase. In addition, we evaluated the DNA damage in the three cell lines by assessing the formation of apurinic/apyrimidinic (AP) sites, one of the major types of DNA lesions. We discovered that the administration of 1 nM and 5 nM TRB and LUR resulted in a significantly higher number of AP sites in all cell lines tested, in a dose-dependent manner, with no overt differences between the two drugs (Supplementary Figure S5).

Overall, the data indicate that TRB and LUR effectively inhibit the growth of iCCA cell lines by limiting proliferation, inducing apoptosis, and causing DNA damage.

### 2.2. Signaling Pathways Affected by Trabectedin and Lurbinectedin in Intrahepatic Cholangiocarcinoma Cell Lines

**Figure 5:**
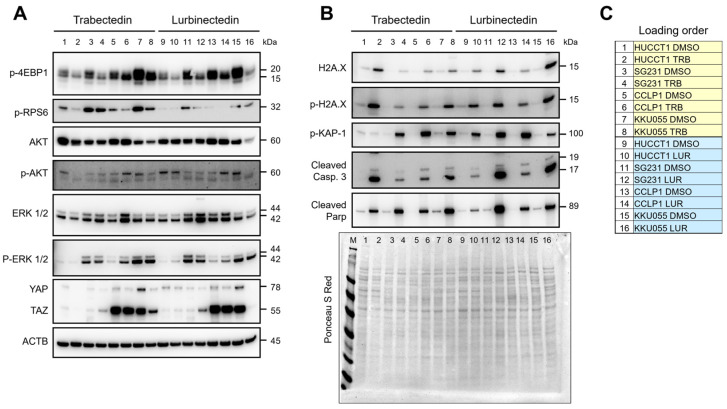
Effects of trabectedin and lurbinectedin on oncogenic cascades, DNA damage response, and apoptosis in intrahepatic cholangiocarcinoma cell lines. Western blot analysis was applied to assess the levels of several effectors of oncogenic cascades (**A**) and DNA damage response and apoptosis pathways (**B**) in HUCCT1, SG231, CCLP1, and KKU055 intrahepatic cholangiocarcinoma (iCCA) cell lines exposed to trabectedin (TRB) and lurbinectedin (LUR) at 1 nM concentration. Cells were treated for 48 h, and Western blot analysis was conducted at this time point. Representative blots are shown. (**C**) Sample loading order. Ponceau S Red staining of the membranes and β-actin were used as a loading control. Abbreviation: M, protein marker.

Next, we aimed to identify the oncogenic cascades modulated by TRB and LUR in iCCA cells. Therefore, protein and mRNA extracted from HUCCT1, SG231, CCLP1, and KKU055 cell lines treated with DMSO, 1 nM TRB, and 1 nM LUR were subjected to Western blot (Figure 5A–C) and quantitative real-time RT-PCR (Supplementary Figures S6 and S7) analyses. Western blotting revealed that TRB and LUR triggered the downregulation of the mammalian target of rapamycin complex 1 (mTORC1) pathway, as assessed by the decrease in its surrogate markers activated/phosphorylated (p)RPS6, and inactivated/phosphorylated (p)4EBP1, whereas the mammalian target of rapamycin complex 2 (mTORC2; as defined by the levels of activated/phosphorylated protein kinase B [AKT]) was not consistently reduced. In addition, the Hippo effector Yes-associated protein 1 (YAP) was downregulated in the cell lines treated with TRB and LUR, whereas the two drugs did not affect the levels of the YAP paralog transcriptional co-activator with PDZ-binding motif (TAZ). In line with the downregulation of YAP protein, the mRNA levels of YAP1 and its specific target genes, connective tissue growth factor (CTGF) and cysteine-rich angiogenic inducer 61 (CYR61), significantly decreased following treatment with TRB and LUR in both the KKU055 and HUCCT1 cell lines. No significant differences were observed between the effects of the two drugs on the levels of these genes (Supplementary Figure S6). In contrast, the two drugs did not influence the extracellular signal-regulated kinase/mitogen-activated protein kinase (ERK/MAPK) pathway (Figure 5A). As expected, the DNA damage markers, including total and activated/phosphorylated histone H2A variant X (H2A.X), as well as activated/phosphorylated KRAB-associated protein 1 (KAP-1), were commonly induced in the four cell lines following TRB and LUR administration, in keeping with the drugs’ role as damaging agents. In addition, the drug treatment remarkably increased the levels of apoptosis markers, cleaved caspase 3, and cleaved Poly (ADP-ribose) polymerase (PARP), in comparison to the low or absent levels of these proteins in DMSO-treated cells (Figure 5B). LUR has been shown to inhibit the expression of oncogenic transcription factors in melanoma cells [37]. Therefore, we investigated the effects of TRB and LUR on the mRNA levels of cellular myelocytomatosis oncogene (*c-Myc*), E2F transcription factor 1 (*E2F1*), TEA domain transcription factor 4 (*TEAD4*), Bromodomain-containing protein 4 (*BRD4*), and cyclin-dependent kinase 7 (*CDK7*) transcription factors, which are either critical in cholangiocarcinogenesis or were downregulated in the referenced study, in KKU055 and HUCCT1 cells. Both drugs decreased the overall levels of these transcription factors, with LUR treatment inducing the most pronounced downregulation in *c-Myc*, *E2F1*, *BRD4*, and *CDK7* genes (Supplementary Figure S7).

Altogether, the present data indicate that TRB and LUR affect various pathways and oncogenic transcription factors in iCCA cells.

### 2.3. Trabectedin and Lurbinectedin Decrease Mitochondrial Respiration and Glycolysis in iCCA Cells

**Figure 6:**
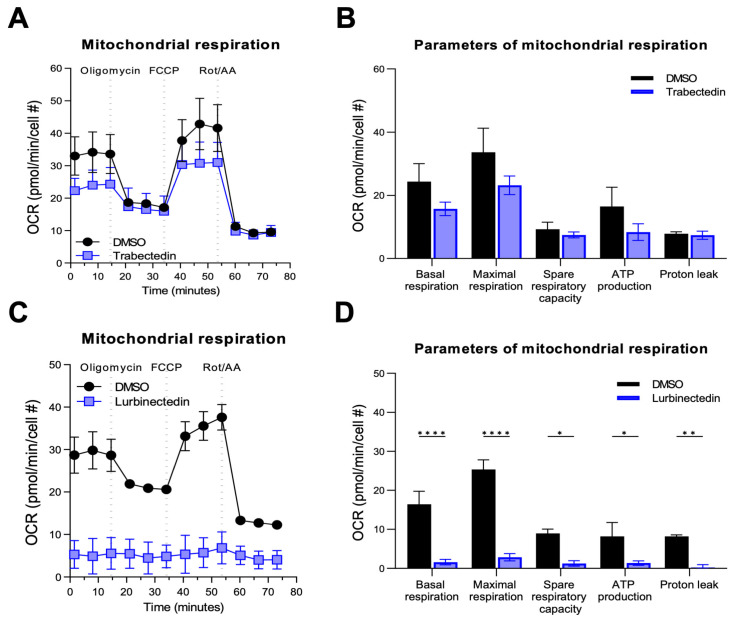
Effect of trabectedin and lurbinectedin on the mitochondrial respiration of human intrahepatic cholangiocarcinoma cells. For the Seahorse XF Mito Stress Test, KKU055 cells were treated with 5 nM trabectedin (TRB), 1 nM lurbinectedin (LUR), or a matched DMSO concentration for 24 h. The Seahorse Mito Stress Test profile of normalized oxygen consumption rate (OCR) in TRB-treated (**A**,**B**) and LUR-treated (**C**,**D**) KKU055 cells is shown. All OCR levels were background corrected and normalized to nuclei fluorescent staining. The mean ± standard deviation of two independent experiments (with technical triplicates) is shown. Dotted lines indicate the time point of compound injection. Cell #, cell number; FCCP, carbonyl cyanide-p-trifluoromethoxyphenylhydrazone; Rot/AA, rotenone/antimycin A. Data are presented as mean ± SD and were analyzed using Tukey’s multiple comparisons test (* *p* < 0.05, ** *p* < 0.01, **** *p* < 0.0001).

To evaluate whether TRB and LUR affect iCCA metabolism, we assessed their influence on mitochondrial respiration and glycolysis of KKU055 and HUCCT1 cells. To determine the mitochondrial respiratory capacity, the oxygen consumption rate was quantified (Figure 6). In KKU055 cells, LUR significantly reduced the cell line’s mitochondrial respiration parameters (basal and maximal respiration, spare respiratory capacity, ATP production, and proton leak (Figure 6C,D). No effects of TRB on mitochondrial respiration were observed at 1 nM concentration. A similar trend to that observed for LUR was detected when TRB was administered at 5 nM concentration, although the data did not reach statistical significance (Figure 6A,B). Similar results were obtained in HUCCT1 cells.

**Figure 7:**
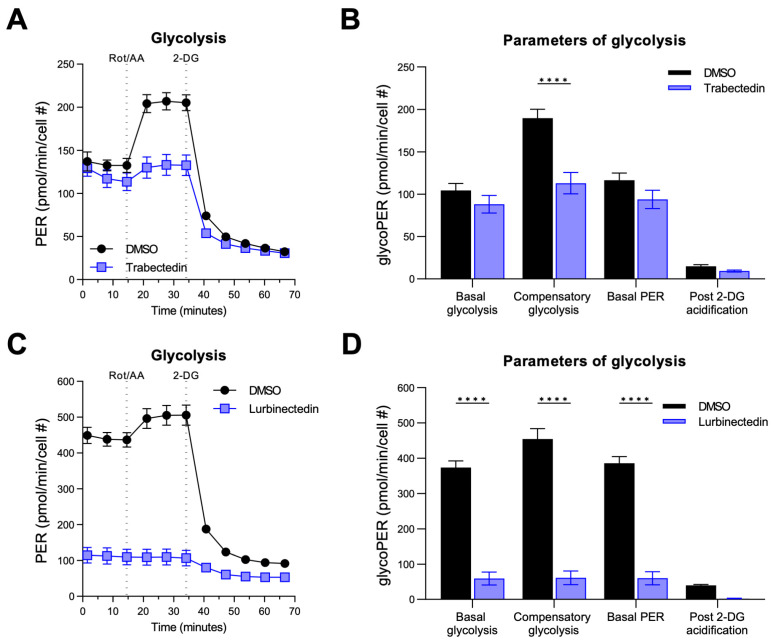
Effect of trabectedin and lurbinectedin on glycolysis of human intrahepatic cholangiocarcinoma cells. For the Seahorse XF Glycolytic Rate Assay, KKU055 cells were treated with 5 nM trabectedin (TRB; (**A**,**B**)), 1 nM lurbinectedin (LUR; (**C**,**D**)), or a matched DMSO concentration for 24 h. Extracellular acidification rates (ECAR) and oxidative stress rates (OCR) were measured in the Seahorse XF HS mini analyzer and converted to proton efflux rate (PER) using the WAVE Pro software (version 10.1.0.1). The first three measurements show basal respiration, followed by injection of rotenone/antimycin A (Rot/AA) and 2-deoxy-D-glucose (2-DG) after the third and sixth measurement, respectively. The data are expressed as mean ± standard deviation (SD) of two independent experiments in triplicate. Right: Measures of basal and compensatory glycolysis calculated from the OCR traces (mean ± SD of two independent experiments; **** *p* < 0.0001, Tukey’s multiple comparisons test). Dotted lines indicate the time point of compound injection. Cell #, cell number.

As concerns glycolysis, both drugs affected this metabolic pathway in KKU055 cells (Figure 7). Once again, LUR administration significantly decreased most parameters (basal and compensatory glycolysis, and basal proton efflux rate) (Figure 7C,D), whereas TRB reached statistical significance only in terms of compensatory glycolysis at 5 nM concentration (Figure 7A,B). Similar data were obtained in HUCCT1 cells.

To investigate further the molecular mechanisms by which TRB and LUR influence mitochondrial respiration and glycolysis, we evaluated the expression levels of key regulators involved in these metabolic processes in KKU055 and HUCCT1 cells. We focused on some regulators that are governed by the transcription factors E2F1 and c-Myc, which we found to be downregulated by TRB and LUR. Our results indicated that mitochondrial transcription factor A (TFAM) and nuclear respiratory factor 1 (NRF1; both involved in mitochondrial biogenesis), as well as lactate dehydrogenase A (LDHA) and phosphoglacerate mutase 1 (PGAM1; both involved in glycolysis), were all downregulated in both KKU055 and HUCCT1 cell lines, particularly in the cells treated with LUR (Supplementary Figure S8).

Overall, the data suggest that LUR and, to a lesser extent, TRB reduce mitochondrial respiration and glycolysis of iCCA cells.

### 2.4. Effects of Trabectedin and Lurbinectedin on the Subcellular Structure of Intrahepatic Cholangiocarcinoma Cells

**Figure 8:**
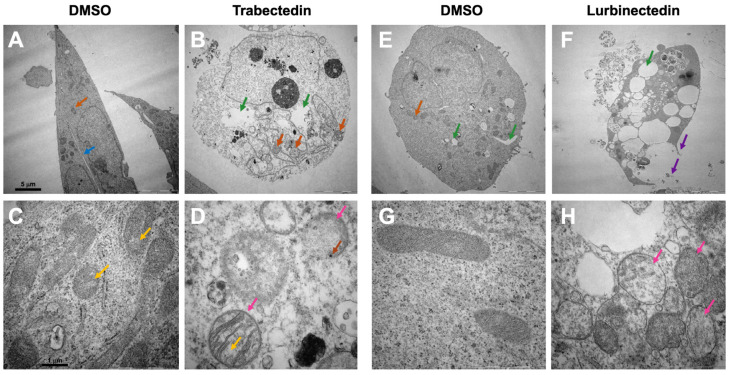
Trabectedin and lurbinectedin severely alter the ultrastructural morphology of intrahepatic cholangiocarcinoma cells. Representative results for the analysis of cell morphology via transmission electron microscopy. KKU055 cells were treated with 1 nM trabectedin (TRB) or 1 nM lurbinectedin (LUR) and compared to a DMSO control. (**A**) DMSO control, whole cell. (**B**) TRB-treated, whole cell. (**C**) DMSO control, mitochondria. (**D**) TRB-treated, mitochondria. The orange arrow indicates mitochondria, whereas the blue arrow shows the fine line separating two cells. Hollow spaces are highlighted with a green arrow. Marked with a pink arrow are mitochondria in the TRB-treated cells, which appear swollen. The yellow arrow indicates irregularities in cristae and the density of mitochondrial matrix. The brown arrow highlights black dots, suggesting possible calcium aggregation. (**E**) DMSO control, whole cell. (**F**) LUR-treated, whole cell. (**G**) DMSO control, mitochondria. (**H**) LUR-treated, mitochondria. The orange arrow indicates mitochondria, while the green arrow highlights hollow spaces, which are strikingly enlarged in the LUR-treated cell. The loss of membrane integrity and the leakage of cell contents in the LUR-treated cell are marked with purple arrows. Highlighted with pink arrows are mitochondria, which are swollen. Ten cells per group were imaged, and exemplary representative images (**A**,**B**,**E**,**F**) are shown at 5000× magnification. Scale bar = 5 µm. Exemplary representative images (**C**,**D**,**G**,**H**) are shown at 40,000× magnification. Scale bar = 1 µm.

Next, the ultrastructural morphology of iCCA cells from KKU055 treated with TRB and LUR was analyzed using transmission electron microscopy. Various parameters were examined to assess changes in cell morphology, including cell size and shape, the status of the nucleus (heterochromatin and euchromatin), and the state of the mitochondria (size, shape, matrix density, and cristae structure). After treatment with the drugs, several notable changes were observed. Membrane blebbing and the presence of apoptotic bodies indicated the onset of apoptosis. Additionally, many cells appeared rounded, making it challenging to distinguish between heterochromatin and euchromatin. Also, the mitochondria exhibited significant alterations, such as a less electron-dense matrix and irregularities in the cristae structure. Some mitochondria showed a rounded shape and pronounced swelling. Furthermore, vacuole formation was observed, along with partial rupture of the cell membrane. These findings were consistent following exposure to either TRB or LUR (Figure 8).

### 2.5. Trabectedin and Lurbinectedin Hamper Intrahepatic Cholangiocarcinoma Organoid Growth

**Figure 9:**
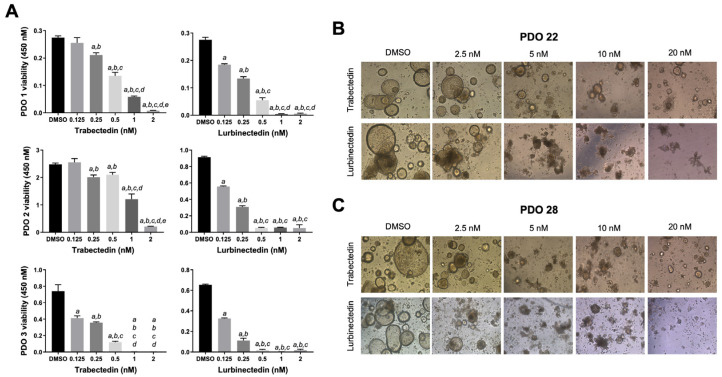
Effects of trabectedin and lurbinectedin on the viability and growth of patient-derived intrahepatic cholangiocarcinoma organoids. (**A**) MTT cell viability assay of patient-derived organoids 1 (PDO1), PDO2, and PDO3 treated for five days with increasing concentrations of trabectedin (TRB) and lurbinectedin (LUR; 0.125, 0.25, 0.5, 1, and 2 nM). Organoids treated with solvent (DMSO) served as controls. Results are expressed as mean ± standard deviation of three independent experiments in triplicate. For statistical analysis, Tukey’s multiple comparisons test was performed; at least *p* < 0.001; a, vs. DMSO, b, vs. 0.125 nM TRB or LUR; c, vs. 0.25 nM TRB or LUR; d, vs. 0.5 nM TRB or LUR; e, vs. 1 nM TRB or LUR. (**B**,**C**) Microscopic images demonstrate that treatment with TRB and LUR resulted in a reduction in the compact, well-organized structure of the organoids, evidenced by the loss of the typical cystic shape associated with cholangiocarcinoma organoids. The PDO22 (upper panels) and PDO28 (lower panels) are shown. Representative brightfield (BF) images (10× magnification) of PDOs are reported.

Organoids are three-dimensional miniature structures derived from stem cells that recapitulate the cellular heterogeneity, structure, and functions of human organs, often serving as a drug testing model [38,39]. A total of six organoids derived from iCCA tumor specimens, referred to as PDO1, PDO2, PDO3, PDO4, PDO22, and PDO28, were obtained from tumor resections performed at the Careggi University Hospital (Florence, Italy) and the Istituto Tumori “Giovanni Paolo II” (Bari, Italy). After treating these organoids with TRB and LUR for 72–120 h, we observed a significant decrease in organoid survival and notable morphological changes as the drug concentrations increased (Figure 9). Overall, at the same drug concentration, the anti-growth effect induced by LUR was more pronounced than that of TRB across the organoids tested.

### 2.6. Trabectedin and Lurbinectedin Hinder Cancer-Associated Fibroblast Growth In Vitro

**Figure 10:**
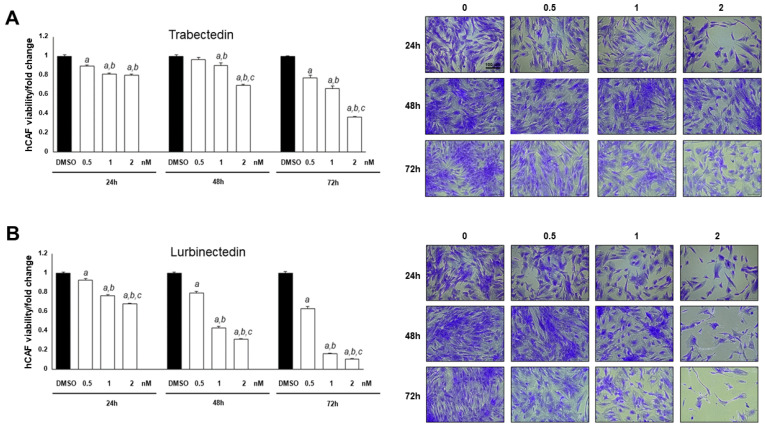
Trabectedin and lurbinectedin restrain the growth of intrahepatic cholangiocarcinoma human cancer-associated fibroblasts. Decrease in intrahepatic cholangiocarcinoma (iCCA), human cancer-associated fibroblasts (hCAF) viability following trabectedin (TRB, (**A**)) or lurbinectedin (LUR, (**B**)) administration. The effect of increasing TRB and LUR concentrations (ranging from 0.5 to 2 nM) on the viability of iCCA hCAFs, grown for 24, 48, and 72 h in culture medium supplemented with 10% FBS, is shown. Experiments were conducted twice in triplicate. At least *p* < 0.001 calculated with One-way ANOVA test; a, vs. DMSO; b, vs. 0.5 nM TRB or LUR; c, vs. 1 nM TRB or LUR. Original magnification: 10×; scale bar: 100 µm.

Accumulating evidence from other tumor types suggests that TRB and LUR play a role in regulating the tumor microenvironment [22,23,33,34,40]. Therefore, we investigated the effects of these two compounds on the growth of human primary cultures of cancer-associated fibroblasts (hCAFs) isolated from patients with iCCA (Figure 10). Specifically, we conducted drug treatments at increasing concentrations and assessed cell viability at three different time points: 24, 48, and 72 h. Notably, we observed a dose- and time-dependent decrease in hCAF viability for both drug treatments, with the lowest cell viability recorded following treatment with LUR (Figure 10). Thus, TRB and LUR may also exhibit anti-oncogenic properties by suppressing the growth of hCAFs.

### 2.7. Lurbinectedin Reduces iCCA Growth In Vivo

**Figure 11:**
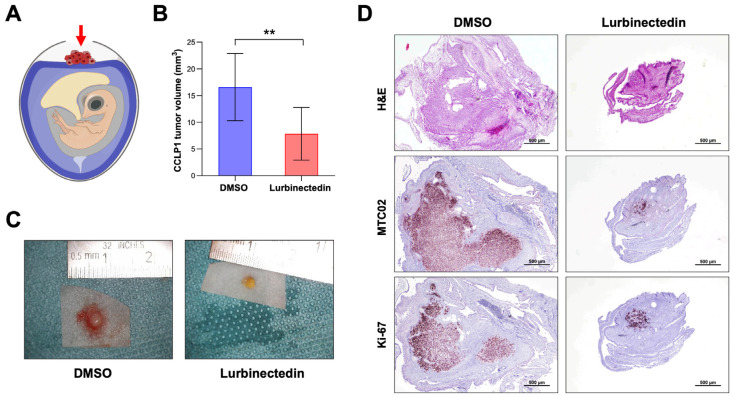
Lurbinectedin reduces the growth of CCLP1 intrahepatic cholangiocarcinoma cells in the chicken chorioallantoic membrane in vivo model. (**A**) Chorioallantoic membrane (CAM) assay scheme. The implanted tumor cells are indicated by a red arrow. Tumor cell-Matrigel^®^ xenografts implanted topically onto the CAM into a silicon ring, were treated either with DMSO or 40 nM lurbinectedin (LUR) and harvested on day 5 after implantation. (**B**) Mean tumor volumes. DMSO (*n* = 11) and LUR-treated (*n* = 14). Student’s *t*-test: ** *p* < 0.01. (**C**) Representative ex ovo macroscopic images of xenograft tumors treated with DMSO or with 40 nM LUR. (**D**) Representative images of hematoxylin and eosin (H&E), MTC09, and Ki-67-stained paraffin sections of CAM tumors. Original magnification: 20×; scale bar: 500 µm.

Finally, we assessed the impact of LUR on the growth of iCCA in vivo using the chick chorioallantoic membrane (CAM) assay (Figure 11). For this experiment, we selected CCLP1 cells, as they proliferate well in the CAM environment. These cells were transplanted onto the CAM, treated with LUR at a concentration of 40 nM, and cultured in ovo for 5 days (Figure 11A). In line with the in vitro data, tumors that developed from LUR-treated cell samples (*n* = 14) were significantly smaller than those treated with DMSO (*n* = 11), measuring 16.6 ± 6.7 mm^3^ vs. 7.9 ± 4.2 mm^3^, respectively (*p* < 0.001) (Figure 11B,C). The smaller tumor size in specimens treated with LUR was confirmed through immunohistochemistry using the anti-MTC02 antibody, which recognizes an antigen specifically associated with mitochondria in human cells (Figure 11D) [41].

## 3. Discussion

iCCA is an aggressive epithelial malignancy of the biliary tract characterized by increasing global incidence and late-stage clinical presentation owing to the lack of disease-specific symptoms. The tumor exhibits marked therapeutic resistance, rapid progression, and poor patient survival, rendering it a major oncological challenge. Consequently, iCCA constitutes a significant health burden worldwide, underscoring the urgent need for innovative therapeutic strategies to improve clinical outcomes in this lethal cancer type [1,2,3,4,5].

In this study, we investigated the effects of TRB and LUR on the growth of iCCA cells. Both TRB and LUR are promising antineoplastic agents known for their favorable toxicity profiles and unique mechanisms of action. These drugs create adducts in the minor groove of DNA, leading to single-strand and double-strand breaks. This process initiates a cascade of events that ultimately results in cell cycle arrest and apoptosis [16,17,18]. Moreover, TRB and LUR mediate the displacement of oncogenic transcription factors from their target promoters, thereby affecting oncogenic signaling addiction [25,26,37]. In clinical settings, TRB is used as a second-line treatment for liposarcoma, leiomyosarcoma, and relapsed platinum-sensitive ovarian cancer, while LUR is administered for metastatic small-cell lung cancer [27,28,29,30].

Regarding iCCA, only one promising study has examined the effects of TRB, which was found to inhibit genes and microRNAs related to several cellular processes, including protein modification, migration, motility, and apoptosis, in a patient-derived xenograft and associated cell line [42]. To date, the effects of LUR in this tumor entity have not been explored. In the present study, we systematically evaluated the growth-inhibitory properties of TRB and LUR using a spectrum of in vitro and in vivo models of biliary tract cancer. We demonstrate that both agents markedly suppress the proliferation of human iCCA cell lines, spheroids, PDOs, and hCAFs, as well as tumor growth in the chicken CAM model.

Notably, we observed that the restraint of tumor cell growth and the inhibition of hCAFs occurred at low nanomolar concentrations of both drugs, highlighting their effectiveness in the tested models. These results indicate that TRB and LUR effectively target both cancer cells and the tumor microenvironment (TME) in iCCA. Supporting this observation, previous studies have shown that TRB and LUR possess the unique ability to simultaneously kill cancer cells while affecting several aspects of the TME across various cancer types. The drugs achieve this objective by inducing selective apoptosis of monocytes and macrophages and by inhibiting the transcription of multiple inflammatory mediators, which ultimately helps alleviate the immunosuppressive environment [33,40]. As the models used in this study do not include the immune component, further investigations are necessary to clarify how TRB and LUR affect the immune milieu in biliary tract cancers. Our investigation also included tumor organoids derived from several patients with iCCA. We found that LUR achieved significant growth inhibition at a lower dosage compared to TRB in these models, highlighting the potential of both drugs against this aggressive disease. At the molecular level, the observed reduction in iCCA cell proliferation, the induction of DNA damage, and the increased apoptosis were associated with the downregulation of the mTORC1 and YAP pathways, which are crucial in cholangiocarcinogenesis [43]. Additionally, both drugs, particularly LUR, significantly lowered the expression of proto-oncogenes such as *YAP1*, *TEAD4*, *E2F1*, *c-Myc*, *BRD4*, and *CDK7* in iCCA cells, aligning with their inhibitory effects on oncogenic transcription factors [43].

In the context of cellular metabolism, treatment with LUR resulted in a significant decrease in mitochondrial respiration and glycolysis in iCCA cell lines. This pattern was also observed with TRB, although to a lesser extent. Ongoing work in our laboratory is focused on delineating the specific targets of TRB and LUR in iCCA growth and metabolic pathways.

While this study presents promising findings that may inform future treatment strategies for iCCA, several limitations should be considered. Although a broad range of preclinical models was employed, including human iCCA cell lines, 3D spheroids, PDOs, CAFs, and the CAM assay, the lack of validation in mammalian models limits conclusions regarding pharmacokinetics, systemic toxicity, and tolerability. The CAM assay, while offering an in vivo-like environment and not classified as an animal model under EU regulations, does not replicate the complexity of mammalian immune and stromal interactions, which are essential for evaluating drug responses in a clinically relevant context. Also, the study did not investigate long-term treatment effects or the emergence of resistance mechanisms, which are particularly relevant given the aggressive nature and therapeutic resistance of iCCA. Additionally, the limited number of PDOs used may not fully reflect the molecular and phenotypic heterogeneity of the disease, potentially affecting the generalizability of the findings. Our assays, including Annexin V-PE/7-AAD flow cytometry, cleaved PARP and caspase-3 Western blotting, γH2AX detection, and BrdU incorporation, were designed to assess apoptosis, necrosis, and DNA damage. However, other regulated cell death modalities such as immunogenic cell death (ICD) and ferroptosis were not evaluated. These pathways require distinct readouts, such as calreticulin exposure, extracellular ATP and HMGB1 release for ICD, and iron-dependent lipid peroxidation and ROS accumulation for ferroptosis. Previous studies have shown that LUR can induce ICD and that TRB may trigger ferroptosis and modulate tumor-associated macrophages [44,45], suggesting that additional mechanisms may contribute to the observed antitumor effects. Finally, molecular markers need to be identified to determine which patients may benefit from the administration of LUR and TRB. Current data suggest that iCCA cell lines are highly sensitive to these compounds, regardless of their mutation profiles. These findings suggest that malignant cholangiocytes are inherently vulnerable to TRB. However, further investigation using a broader range of experimental models is needed to determine whether specific genetic alterations contribute to susceptibility or resistance to these drugs in biliary tumors. In addition, large-scale transcriptomic and proteomic approaches across various cancer types should be conducted to determine whether the molecular markers of susceptibility/resistance to TRB and LUR are common among different cancer entities or are tumor-specific.

Future research should aim to confirm the in vivo efficacy of TRB and LUR in mammalian models and explore their impact on the immune microenvironment. Moreover, investigating combination therapies with established or emerging agents may enhance therapeutic efficacy and help overcome resistance. Furthermore, expanding the diversity of patient-derived models and incorporating immune-competent systems will be essential to validate these strategies and support their clinical translation.

## 4. Materials and Methods

### 4.1. Cell Lines and Reagents

The HUCCT1, KKU156, KKU213, CCLP1, KKU055, and SG231 human iCCA cell lines, purchased from the Japanese Collection of Research Bioresources (JCRB; Ibaraki, Osaka, Japan) or the American Type Culture Collection (ATCC; Manassas, VA, USA), were employed in the experimental procedures. CCLP1, KKU055, KKU156, KKU213, and SG231 cells were grown in Dulbecco’s modified Eagle medium (Gibco, Grand Island, NY, USA), whereas HUCCT1 cells were grown in RPMI 1640 medium (Gibco). All media were supplemented with 5% fetal bovine serum (Gibco), 100 mg/mL streptomycin, and 100 U/mL penicillin, 10 mM HEPES, 2 mM L-glutamine, and 1 mM sodium pyruvate (Anprotec, Milpitas, CA, USA). Cells were cultured at 37 °C in a 5% CO_2_ humidified atmosphere, and mycoplasma-free status for all cell lines was recurrently tested using the PCR Mycoplasma Test Kit I (PK-CA91-1096, PromoCell, Heidelberg, Germany). Authentication of cell lines was conducted by Cell Lines Service (Eppelheim, Germany). The drugs TRB (HY-50936, MedChemExpress, Junction, NJ, USA) and LUR (HY-16293, MedChemExpress) were used in the experiments. Stock solutions (1 and 10 mM) were prepared in dimethylsulfoxide (DMSO), and aliquots were stored at −20 °C.

### 4.2. Viability, Proliferation, Apoptosis, and DNA Damage Assays

iCCA cells were seeded in 96-well plates at a density of 1.0 × 10^4^ cells per well and exposed to various concentrations of TRB and LUR for 48 h. Cells treated with the solvent (DMSO) and wells containing only culture medium were used as negative and background controls, respectively. Concerning the MTT assay, following treatment, 10 μL of 5 mg/mL methyl-thiazolyl-diphenyltetrazolium bromide (MTT) solution was added per 96 well and incubated at 37 °C and under 5% CO_2_ for 2 h. The medium was completely removed, and 100 μL of 100% (*v*/*v*) DMSO was added per well to dissolve the formazan crystals, and the absorbances were measured at 570 and 630 nm using the FLUOstar Omega multiplate reader and software version 5.50 R4 (BMG LABTECH, Ortenberg, Germany), and MARS data analysis software version 3.32 R5 (BMG Labtech, Ortenberg, Germany). The average absorbance of the DMSO-treated cells was established as 100% cell viability. IC_50_ values and standard deviations were calculated using the GraphPad Prism software version 9.5.1 (GraphPad Software Inc., San Diego, CA, USA). Data from three independent experiments, each with eight wells per condition, were combined.

Cell proliferation was assessed at the 48 h time point using the BrdU Cell Proliferation Assay Kit (Cell Signaling Technology, Danvers, MA, USA). Briefly, cells were incubated with 1x bromodeoxyuridine (BrdU) for 2 h and fixed for 30 min at room temperature. Subsequently, the fixing solution was discarded, and cells were incubated with the anti-BrdU mouse antibody (# 5292; Cell Signaling Technology) for 1 h at room temperature. After washing, cells were stained with the HRP-conjugated anti-mouse secondary antibody for 30 min at room temperature. After incubation with the TMB substrate solution for an additional 30 min at room temperature, the stop solution was added, and the absorbance was measured at 450 nm.

Apoptosis was determined in the iCCA cell lines using the Cell Death Detection ELISA Plus Kit (Roche Molecular Biochemicals, Indianapolis, IN, USA), following the manufacturer’s protocol. To assess the extent of DNA damage, the DNA Damage Assay Kit (Abcam, Cambridge, UK) was applied following the manufacturer’s instructions. Cell line experiments were repeated at least three times in triplicate.

### 4.3. Flow Cytometry Assay

Flow cytometry analysis was performed after treating iCCA cells with TRB or LUR. Briefly, cells were seeded in 6-well plates at a density of 2.5 × 10^5^ cells per well, and then cultured in the presence of TRB or LUR at 1 nM concentration. After 24 h, cells and media were recovered, pelleted, and washed with PBS before staining with live/dead cell marker 7-aminoactinomicyn-D (7AAD, BD Biosciences, San Jose, CA, USA) and further resuspension in 1x Annexin V binding buffer containing Annexin V (Annexin V-PE Apoptosis Detection Kit; BD Biosciences). For cell cycle analysis, cells were recovered, washed with PBS, and the pellet solubilized in 70% ethanol and stored at −20 °C overnight. The samples were then stained with 7AAD and incubated for 15 min at room temperature before acquisition. iCCA cells were analyzed using a flow cytometer FACS CANTO II (BD Biosciences), and approximately 30,000 events for each sample were acquired, and data were analyzed using the ModFIT LT 6.0 software (Verity Software House, Topsham, ME, USA) and the Diva 6.2 software (BD Biosciences). Data from three independent experiments were then plotted using GraphPad Prism 8.4 software, and values are presented as mean  ±  standard deviation (SD) of the percentage.

### 4.4. Spheroid Generation and Assessment of Cell Viability

To establish three-dimensional (3D) cultures enriched in CCA stem-like cells, cells were cultured for seven days under non-adherent conditions in poly-2-hydroxyethyl methacrylate (poly-HEMA)-coated dishes (Sigma Aldrich, St. Louis, MO, USA) using serum-free DMEM/F12 medium. The medium was supplemented with 1x B27 supplement (without vitamin A) (Life Technologies, Carlsbad, CA, USA), 20 ng/mL epidermal growth factor (EGF), and 20 ng/mL basic fibroblast growth factor (bFGF) (R&D Systems, Minneapolis, MN, USA), as previously described [35,36]. 3D cultures were dissociated and seeded in 96-well plates for 24 h before treatment with TRB and LUR, at varying concentrations, as detailed in the results section, for an additional 48 h. Images were captured in bright field at 10× magnification. Then, we proceeded to remove the medium, and cells were stained with a 0.5% Crystal Violet solution in 20% methanol for 10 min. After staining, the cells were washed with phosphate-buffered saline (PBS) and solubilized using 100 μL/well of dimethyl sulfoxide (DMSO). The absorbance at 595 nm was measured using a HiPo Biosan microplate reader (Bio Class, Pistoia, Italy).

### 4.5. Patient-Derived Tumor Organoids (PDOs)

PDO cultures were generated following established protocols. Briefly, iCCA tumor specimens (~1 cm^3^) were obtained from surgical resections at the Careggi University Hospital (Florence, Italy) and the Istituto Tumori “Giovanni Paolo II” (Bari, Italy). Samples were transported on ice and processed within 20 min of collection. Tumor tissues were finely minced and incubated with a digestion solution at 37 °C for 2 to 5 h (or overnight), depending on the extent of liver fibrosis. Following digestion, cell clusters were embedded in Matrigel^®^ Growth Factor Reduced (GFR) (Corning, Glendale, CA, USA). After polymerization of the Matrigel, a specific isolation medium was added and maintained until the first passage. Tumor organoids were visually assessed for growth after 2 to 3 weeks. The isolation medium was then replaced with an expansion medium for long-term culture [46].

Cell viability in PDOs was assessed using the MTT assay. Briefly, 1.0 × 10^4^ cells per well were seeded in 96-well plates within 7 μL Matrigel^®^ droplets, incubated for seven days, and treated for 72 h with TRB and LUR (0, 2.5, 5, 10, and 20 nM). MTT solution was added at a final concentration of 500 μg/mL, and organoids were incubated for 2 to 3 h at 37 °C. Thereafter, the medium was removed, and 20 μL of 2% SDS solution in water was added for 2 h at 37 °C to solubilize the Matrigel. Next, 100 μL of DMSO was added to dissolve the reduced MTT. Absorbance was measured at 568 nm using a HiPo Biosan microplate reader (Biosan, Riga, Latvia). Untreated organoids were considered 100% viable [38,39].

### 4.6. Isolation and Treatment of Human Cancer-Associated Fibroblasts (hCAFs)

hCAFs were isolated from iCCA tissues as previously reported [38,47]. iCCA tissue fragments were subjected to enzymatic and mechanical dissociation in HBSS containing 50–200 U/mL collagenase type IV (Thermo Fisher Scientific, Waltham, MA, USA), 3 mM CaCl_2_, and 1× Antibiotic-Antimycotic (Thermo Fisher Scientific). The digestion was performed at 37 °C under gentle rotation for at least 2 h, or longer if necessary. The obtained cell population was collected and washed three times with HBSS by centrifugation. Subsequently, the cell suspension was divided into two parts: one part to obtain organoids and the second for CAFs. As for the culture of hCAFs, after tissue digestion and cell pellet washing were completed, the recovered cell suspension was cultured in IMDM (Iscove’s Modified Dulbecco’s Medium) complete with 20% fetal bovine serum (FBS, Thermo Fisher Scientific) and Antibiotic-Antimycotic. Cultures of iCCA hCAFs were treated with vehicle and increasing concentrations of TRB and LUR. The viability of iCCA hCAF was determined with the CyQUANT™ XTT Cell Viability kit (Thermo Fisher Scientific) at three experimental time points (24, 48, 72 h). Data from three independent experiments were expressed as mean ± SD.

### 4.7. RNA Extraction and Quantitative Real-Time Reverse Transcriptase-Polymerase Chain Reaction (qRT-PCR)

Total mRNA was extracted from iCCA cell lines using the Quick RNA Miniprep kit (Zymo Research, Irvine, CA, USA). Subsequently, mRNA expression of the genes of interest was assessed by quantitative real-time polymerase chain reaction (qRT-PCR). The following TaqMan gene expression assays (Thermo Fisher Scientific) were used: BRD4 (Hs04188087_m1), CDK7 (Hs00361486_m1), CTGF (Hs01026927_g1), CYR61 (Hs00155479_m1), E2F1 (Hs00153451_m1), LDHA (Hs01378790_g1), NRF1 (Hs00602161_m1), PGAM1 (Hs01652468_g1), TEAD4 (Hs01125032_m1), and TFAM (Hs00273372_s1). PCR reactions were conducted using 100 ng of cDNA from the collected samples, with an ABI Prism 7000 Sequence Detection System with TaqMan Universal PCR Master Mix (Applied Biosystems, Waltham, MA, USA). Cycling conditions were as follows: denaturation at 95 °C for 10 min, 40 cycles at 95 °C for 15 s, and then extension at 60 °C for 1 min. Quantitative values were calculated using the PE Biosystems Analysis software version 1.2.3 and expressed as N target (NT). NT  =  2^−ΔCt^, where each sample’s ΔCt value was calculated by subtracting the average Ct value of the target gene from the average Ct value of the β-actin gene (4333762T; Thermo Fisher Scientific).

### 4.8. Protein Extraction and Western Blot Analysis

Cells were homogenized in M-PER Mammalian Protein Extraction Reagent (Thermo Fisher Scientific) containing the Complete Protease Inhibitor Cocktail (Roche Molecular Biochemicals, Indianapolis, IN, USA). Protein concentrations were determined with the Bio-Rad Protein Assay Kit (Bio-Rad, Hercules, CA, USA) using bovine serum albumin as a standard. For Western blotting, 40 μg protein was denatured by boiling in Tris-MOPS-SDS running buffer, resolved using SurePAGE gels (GenScript, Piscataway, NJ, USA), and transferred onto nitrocellulose membranes (Bio-Rad) via electroblotting in Towbin buffer (25 mM Tris, 195 mM glycine, 20% methanol). Membranes were blocked in Pierce Protein-free Tween 20 Blocking Buffer (Thermo Fisher Scientific) for 1 h and probed with the following specific antibodies from Cell Signaling Technology: anti-phospho-4EBP1^Thr37/46^ (#2855), phospho-RPS6^Ser235/236^ (#4858), AKT (#4691), phospho-AKT^Ser473^ (#4060), ERK1/2 (#4695), phospho-ERK1/2^Thr202/Tyr204^ (#4370), YAP/TAZ (#93622), H2A.X (#7631), phospho-H2A.X^Ser139^ (#9718), phospho-KAP-1^Ser824^ (#90893), cleaved Caspase 3 (#9661), cleaved PARP (#5625), and β-Actin (#4970). Each primary antibody was followed by incubation for 30 min with horseradish peroxidase secondary antibody (Jackson ImmunoResearch Laboratories, Inc., West Grove, PA, USA), diluted 1:5000. Equal protein loading was assessed with reversible Ponceau S staining (Thermo Fisher Scientific) and anti-β-Actin antibody. Proteins were revealed with the Clarity Western ECL Substrate (Bio-Rad).

### 4.9. Seahorse Mitochondrial Respiration and Glycolysis Analyses

Seahorse assays on adherent cell lines were performed according to the manufacturer’s instructions. KKU055 and HUCCT1 (1.0 × 10^4^) cells per well were seeded into a Seahorse XFp Cell Culture 8 well Miniplate (Agilent Technologies, Waldbronn, Germany) and cultured overnight at 37 °C and 5% CO_2_ for 24 h in standard culture medium. Two wells with medium only served as background correction. Experiments were conducted using three technical replicates per group. The next day, cells were treated with TRB (5 nM) and LUR (0.7 nM) or matched DMSO concentration in triplicate and incubated for 24 h at 37 °C and 5% CO_2_. One Seahorse XFp sensor cartridge per cell culture plate was hydrated with Seahorse XF Calibrant overnight at 37 °C in a CO_2_-free incubator. On the day of assay, the cells were washed and equilibrated with assay medium (Seahorse XF DMEM Medium pH 7.4, 10 mM glucose, 1 mM sodium pyruvate, and 2 mM L-glutamine) for one hour at 37 °C in a CO_2_-free incubator and transferred to the Seahorse XF HS Mini Analyzer (Agilent Technologies, Waldbronn, Germany). Injection ports were loaded either with 1.5 μM oligomycin (1.5 µM final), FCCP (KKU055, 1 µM final; HUCCT1, 2 µM final), and rotenone/antimycin A (Rot/AA, 0.5 µM final) for the Cell Mito Stress Test or with Rot/AA (0.5 µM final) and 2-deoxy-D-glucose (2-DG; 50 mM final; Sigma-Aldrich, St. Louis, MO, USA) for the Glycolytic Rate Assay. Lastly, Hoechst 33,342 (2 µg/µL final; Thermo Fisher Scientific) nuclei staining was injected per well and incubated for 10 min before fluorometric measurement (excitation, 355-20 nm; emission, 460 nm) using the FLUOStar Omega plate reader and software version 5.50 R4 (BMG Labtech, Ortenberg, Germany). Data were analyzed using the WAVE Pro 10.1.0.1 software (Agilent Technologies). The values were background corrected, normalized to the mean fluorescence intensity (MFI) per well, and a normalization scale factor of 10,000 was applied. Data from three replicates of two independent experiments were used to perform statistical analysis using the GraphPad Prism software version 9.5.1.

### 4.10. Transmission Electron Microscopy Analysis

KKU055 cells were resuspended in Cytoblock Reagent (Epredia, Dreieich, Germany) and subsequently embedded in 4% low-melting agarose. For the embedding process (post-fixation with osmium tetroxide, dehydration, infiltration with EPON) the LYNX microscopy tissue processor (Reichert-Jung, Wetzlar, Germany) was used. Semi-thin sections (0.75 µm), for the selection of relevant areas, and ultra-thin sections (80 nm) were cut by using the Reichert Ultracut S Microtome (Leica-Reichert, Wetzlar, Germany). First, the grids were contrasted with aqueous 2–uranyl-acetate, followed by 2–lead-citrate solution for 10 min, each. Electron-microscopic analysis was performed on 10 cells per group using the LEO 912AB transmission electron microscope (Zeiss, Oberkochen, Germany).

### 4.11. Chicken Chorioallantoic Membrane (CAM) Assay and Immunohistochemical Staining

Fertilized, specific pathogen-free (SPF) chicken eggs (VALO Biomedia, Osterholz-Scharmbeck, Germany) were kept at 37 °C and 80% constant humidity. On day 8, a window of 1.5–2.0 cm diameter was cut in the shell at the more rounded pole of the egg and sealed with tape (Durapore silk tape, 3M). The next day, 2.0 × 10^6^ human tumor cells per pellet were embedded in growth factor-reduced Matrigel (Corning, Wiesbaden, Germany) serving as matrix and were transplanted onto the CAM. The window was sealed again, and the eggs were incubated for 5 days. Tumor progression was monitored over time using the digital microscope camera DigiMicro Profi (9463189,Toolcraft AG, Georgensgmünd, Germany) at 10× magnification. Tumors were sampled with the surrounding CAM on day 5, fixed in 4% formaldehyde, paraffin-embedded, and cut into 3–5 μm sections for immunohistochemical studies. Antigen retrieval was performed by heating samples in 10 mM sodium citrate buffer (pH 6.0) using a microwave at high power for 10 min, followed by a 20 min cool-down at room temperature. After a blocking step with the 5% goat serum and Avidin-Biotin blocking kit (Vector Laboratories, Burlingame, CA, USA), the slides were incubated with primary antibodies overnight at 4 °C. The anti-MTC02 (# MA5-12017; 1:500; Thermo Fisher Scientific) and the anti-Ki-67 antibodies (# IHC-00375; 1:1000; Thermo Fisher Scientific) were applied. Subsequently, slides were treated with 3% hydrogen peroxide for 10 min to quench endogenous peroxidase activity. Next, the biotin-conjugated secondary antibody was applied at a 1:500 dilution for 30 min at room temperature, and the reaction was revealed with the Vectastain^®^ ABC-Elite Peroxidase Kit (# PK-6100; Vector Laboratories), using the ImmPACT NovaRed Substrate Peroxidase (# SK-4805; Vector Laboratories) as the chromogen. Slides were counterstained with Mayer’s hematoxylin.

### 4.12. Statistical Analysis

GraphPad Prism version 10.2.1 software and IBM SPSS version 26 software (IBM Corp., Armonk, NY, USA) evaluated statistical significance. For IC_50_ calculation, data were transformed to log2, normalized, and non-linear regression (log)inhibitor vs. response–variable slope (four parameters) was performed. Tukey’s multiple comparison test was applied for multiple comparisons. Two-tailed values of * *p* < 0.05, ** *p* < 0.01, *** *p* < 0.001, and **** *p* < 0.0001 were considered significant. Lowercase letters denoted statistical significance, as stated in the associated figure legends. All data are expressed as the mean ± SD.

## 5. Conclusions

Overall, our study provides compelling evidence that TRB and LUR exert potent anti-tumor effects in iCCA across a range of preclinical models. Both agents effectively inhibit tumor cell proliferation and target components of the tumor microenvironment, such as CAFs, at low nanomolar concentrations. Mechanistically, TRB and LUR induce DNA damage, apoptosis, and downregulate key oncogenic pathways such as mTORC1 and YAP, while also suppressing the expression of proto-oncogenes and impairing cellular metabolism. These findings highlight the therapeutic potential of TRB and LUR in iCCA and support further investigation into their mechanisms of action and combinatorial strategies. Future studies incorporating mammalian models and immune profiling will be essential to validate these results and advance TRB and LUR toward clinical application in this challenging malignancy.

## Data Availability

All data generated or analyzed during this study are included in this published article (and its Supplementary Information Files).

## Supplementary Materials

The following supporting information can be downloaded at: https://www.mdpi.com/article/10.3390/ijms26189085/s1.

## Abbreviations

5-FU, 5-Fluorouracil; 7-AAD, 7-aminoactinomycin D; AKT, Protein kinase B; AP, apurinic/apyrimidinic sites; BRD4, Bromodomain-containing protein 4; BrdU, Bromodeoxyuridine; CAF, cancer-associated fibroblast; CAM, chorioallantoic membrane; CDK7, Cyclin-dependent kinase 7; c-Myc, cellular Myelocytomatosis oncogene; cleaved PARP, cleaved poly (ADP-ribose) polymerase; CTGF, Connective tissue growth factor; CYR61, Cysteine-rich angiogenic inducer 61; CYP3A4, Cytochrome P450 3A4; DMSO, dimethyl sulfoxide; E2F1, E2F transcription factor 1; EMA, European Medicines Agency; ERK/MAPK, Extracellular signal-regulated kinase/Mitogen-activated protein kinase; FDA, U.S. Food and Drug Administration; FGFR, Fibroblast growth factor receptor; H2A.X, activated/phosphorylated histone H2A variant X; IDH, Isocitrate dehydrogenase; iCCA, Intra-hepatic cholangiocarcinoma; KAP-1, activated/phosphorylated KRAB-associated protein 1; LDHA, Lactate dehydrogenase A; LUR, Lurbinectedin; MASLD, Metabolic dysfunction-associated steatotic liver disease; mTORC1, Mechanistic target of rapamycin complex 1; mTORC2, Mammalian target of rapamycin complex 2; NRF1, Nuclear respiratory factor 1; PDO, Patient-derived tumor organoid; PE, Phycoerythrin; PGAM1, Phosphoglycerate mutase 1; p4EBP1, inactivated/phosphorylated; pRPS6, activated/phosphorylated; SD, Standard deviation; SCLC, Small cell lung cancer; TAZ, Transcriptional co-activator with PDZ-binding motif; TAMs, Tumor-associated macrophages; TEAD4, TEA domain transcription factor 4; TFAM, Mitochondrial transcription factor A; TME, Tumor microenvironment; TRB, Trabectedin; YAP1, Yes-associated protein 1.
