# Supplementary material for: The DNA Minor Groove Binders Trabectedin and Lurbinectedin Are Potent Antitumor Agents in Human Intrahepatic Cholangiocarcinoma
Source: Int J Mol Sci. 2025 Sep 18;26(18):9085. doi: 10.3390/ijms26189085 (PMC12470038; doi:10.3390/ijms26189085)
Supplement: Supplementary file 1 [file ijms-26-09085-s001.zip › ijms-3795877-supplementary.pdf]

Article

The DNA Minor Groove Binders Trabectedin and Lurbinectedin Are Potent Antitumor Agents in Human Intrahepatic Cholangiocarcinoma

Erwin Gäbele [1,2], Isabella Gigante [3], Mirella Pastore [4], Antonio Cigliano [5], Grazia Galleri [6], Thea Bauer [7], Elena Pizzuto [3], Serena Mancarella [3], Martina Müller [1], Fabio Marra [4], Heiko Siegmund [7], Gianluigi Giannelli [3], Matthias Evert [7], Chiara Raggi [4], Diego F. Calvisi [7] and Sara M. Steinmann [7,*]

[1] Department of Internal Medicine I, University Hospital Regensburg, 93053 Regensburg, Germany; gaebele@internisten-regensburg.de (E.G.); martina.mueller-schilling@ukr.de (M.M.)
[2] Internisten-Regensburg.de, Internal Medicine Group Practice, 93053 Regensburg, Germany
[3] National Institute of Gastroenterology, IRCCS "Saverio de Bellis", 70013 Castellana Grotte, Italy; isabella.gigante@irccsdebellis.it (I.G.); elena.pizzuto@irccsdebellis.it (E.P.); serena.mancarella@irccsdebellis.it (S.M.); gianluigi.giannelli@irccsdebellis.it (G.G.)
[4] Department of Experimental and Clinical Medicine, University of Florence, 50121 Florence, Italy; mirella.pastore@unifi.it (M.P.); fabio.marra@unifi.it (F.M.); chiara.raggi@unifi.it (C.R.)
[5] Department of Medicine, Surgery, and Pharmacy, University of Sassari, 07100 Sassari, Italy; acigliano@uniss.it
[6] Department of Biomedical Sciences, University of Sassari, 07100 Sassari, Italy; galleri@uniss.it
[7] Institute of Pathology, University of Regensburg, 93053 Regensburg, Germany; thea.bauer@stud.uni-regensburg.de (T.B.); heiko.siegmund@ukr.de (H.S.); matthias.evert@klinik.uni-regensburg.de (M.E.); diego.calvisi@klinik.uni-regensburg.de (D.F.C.)
* Correspondence: sara.steinmann@ukr.de or sara.steinmann.3012@gmail.com; Tel.: +49-941-944-6721; Fax: +49-941-944-6602

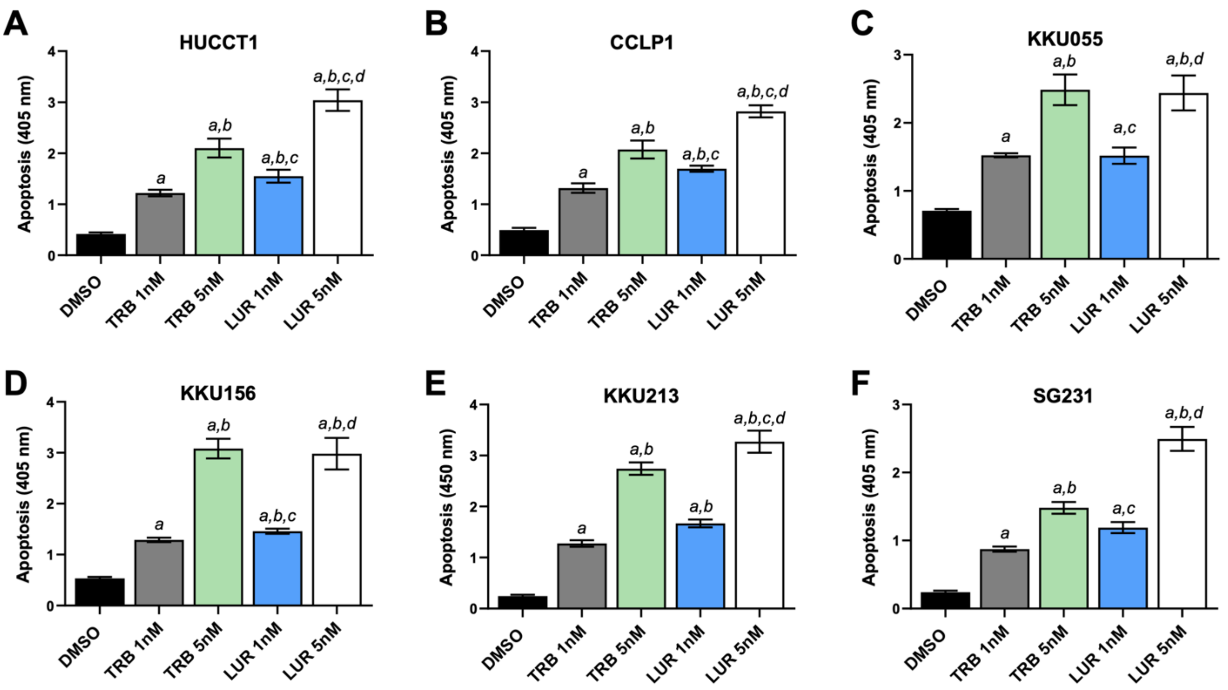

Supplementary Figure S1. Effect of trabectedin and lurbinectedin on the survival of intrahepatic cholangiocarcinoma cell lines. Apoptosis was assessed in HUCCT1 (A), CCLP1 (B), KU055 (C), KKU156 (D), KKU213 (E), and SG231 (F) cell lines treated for 48 hours with trabectedin (TRB) and lurbinectedin (LUR) at 1 nM and 5 nM concentrations. Cells treated with solvent (DMSO) served as controls. All results are expressed as mean ± standard deviation of three independent experiments in triplicate. For statistical analysis, Tukey's multiple comparisons test was performed; at least $p < 0.001$; *a*, vs. DMSO; *b*, vs. 1 nM TRB; *c*, vs. 5 nM TRB; *d*, vs. 1 nM LUR.

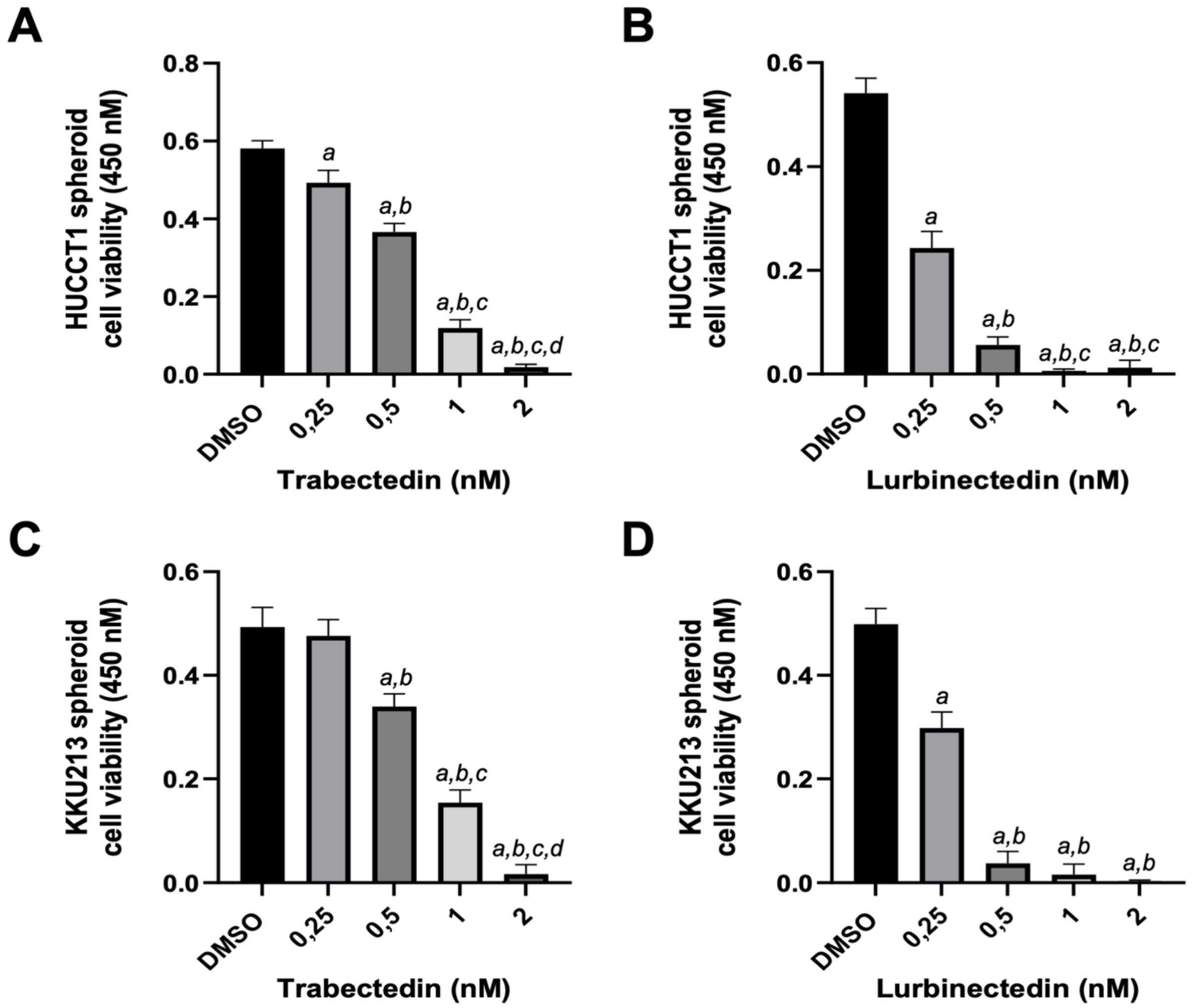

Supplementary Figure S2. Effects of trabectedin and lurbinectedin on the viability of spheroids from HUCCT1 and KKU213 intrahepatic carcinoma cell lines. The two drugs were administered at various concentrations (0.25-2 nM) for five days. Spheroids treated with solvent (DMSO) served as controls. Results are expressed as mean ± standard deviation of three independent experiments in triplicate. For statistical analysis, Tukey's multiple comparisons test was performed; at least $p < 0.001$; *a*, vs. DMSO; *b*, vs. 0.25 nM trabectedin (TRB) or lurbinectedin (LUR); *c*, vs. 0.5 nM TRB or LUR; *d*, vs. 1 nM TRB or LUR.

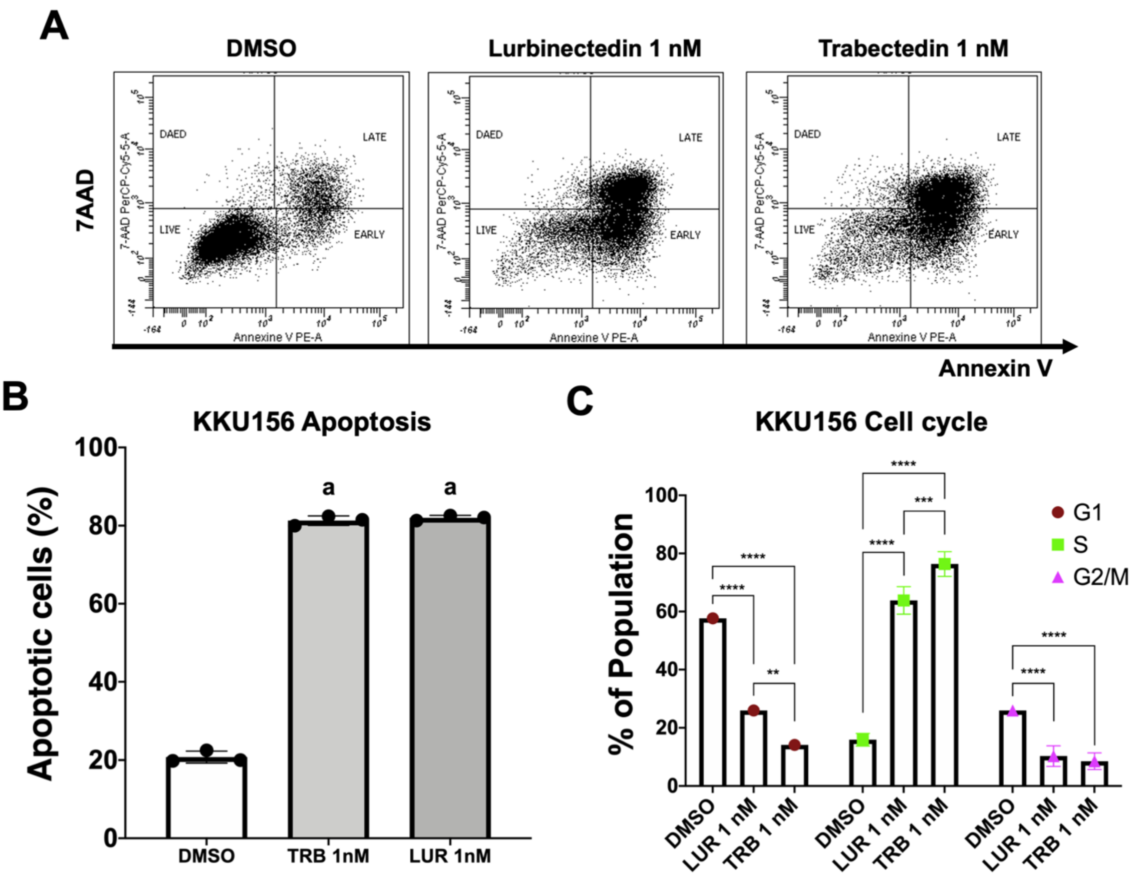

Supplementary Figure S3. Effect of lurbinectedin and trabectedin on the apoptosis and cell cycle of intrahepatic cholangiocarcinoma cell line KKU156, as assessed by flow cytometry analysis. (A,B) Dot plot graph of the apoptotic analysis representative of three independent experiments. Apoptotic analysis with Annexin V-PE and 7-AAD staining of KKU156 intrahepatic cholangiocarcinoma (iCCA) cells treated with trabectedin (TRB) and lurbinectedin (LUR) at 1 nM concentration for 24 hours. Data as the percentage of total apoptotic cells, are presented as mean ± standard deviation (SD), and the significance level of ANOVA is reported (***, $p < 0.001$) according to Tukey's multiple comparisons test. Lowercase letters are used to denote statistical significance (a, vs. vehicle; b, vs. TRB 1 nM). **(C)**. Quantification of the cell cycle phases of iCCA cells that were cultured in complete medium and treated with vehicle or 1 nM LUR or 1 nM TRB for 24 hours from three independent experiments. Data are presented as mean ± SD and were analyzed using Tukey's multiple comparisons test (* $p < 0.01$, *** $p < 0.001$, **** $p < 0.0001$).

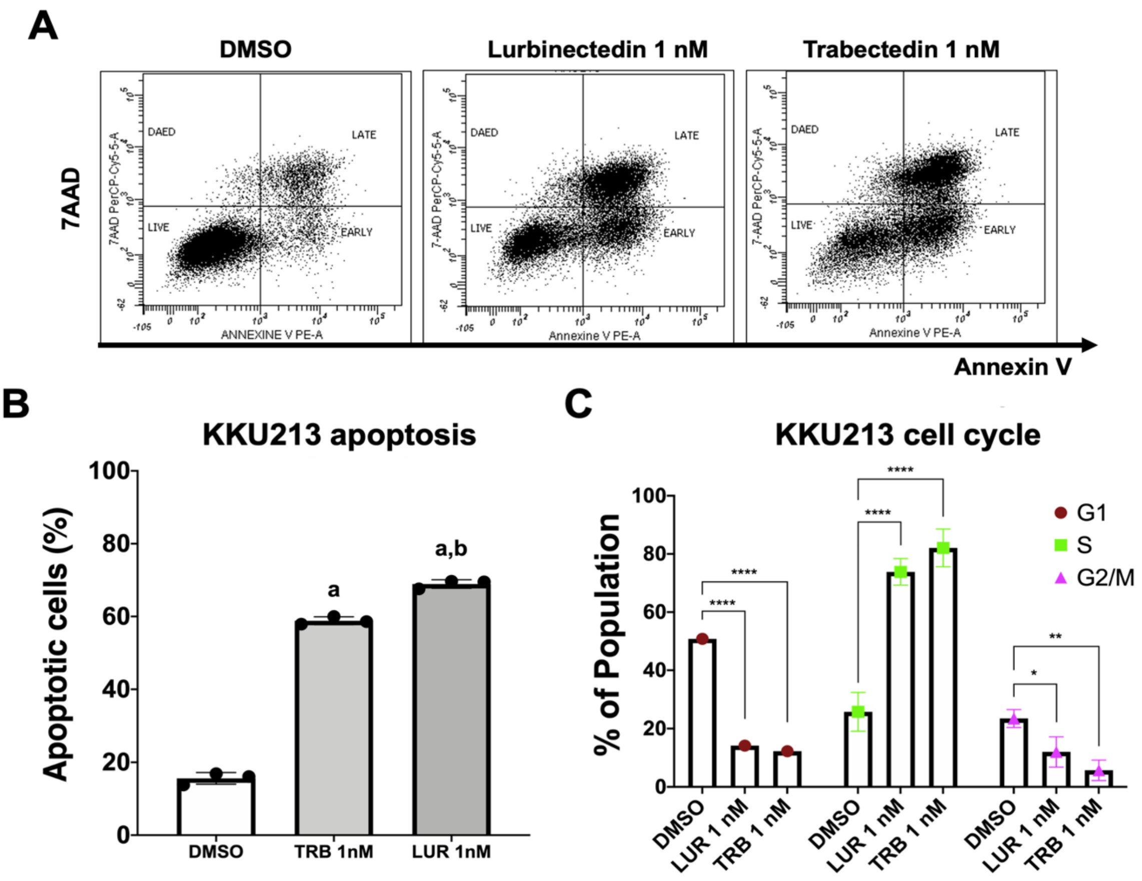

Supplementary Figure S4. Effect of lurbinectedin and trabectedin on the apoptosis and cell cycle of intrahepatic cholangiocarcinoma cell line KKU213, as assessed by flow cytometry analysis. (A,B) Dot plot graph of the apoptotic analysis representative of three independent experiments. Apoptotic analysis with Annexin V-PE and 7-AAD staining of KKU213 intrahepatic cholangiocarcinoma (iCCA) cells treated with trabectedin (TRB) and lurbinectedin (LUR) at 1 nM concentration for 24 hours. Data as the percentage of total apoptotic cells, are presented as mean ± standard deviation (SD), and the significance level of ANOVA is reported (***$p < 0.001$) according to Tukey's multiple comparisons test. Lowercase letters are used to denote statistical significance (a, vs. vehicle; b, vs. TRB 1 nM). **(C)**. Quantification of the cell cycle phases of iCCA cells that were cultured in complete medium and treated with vehicle or 1 nM LUR or 1 nM TRB for 24 hours from three independent experiments. Data are presented as mean ± SD and were analyzed using Tukey's multiple comparisons test (* $p < 0.01$, *** $p < 0.001$, **** $p < 0.0001$).

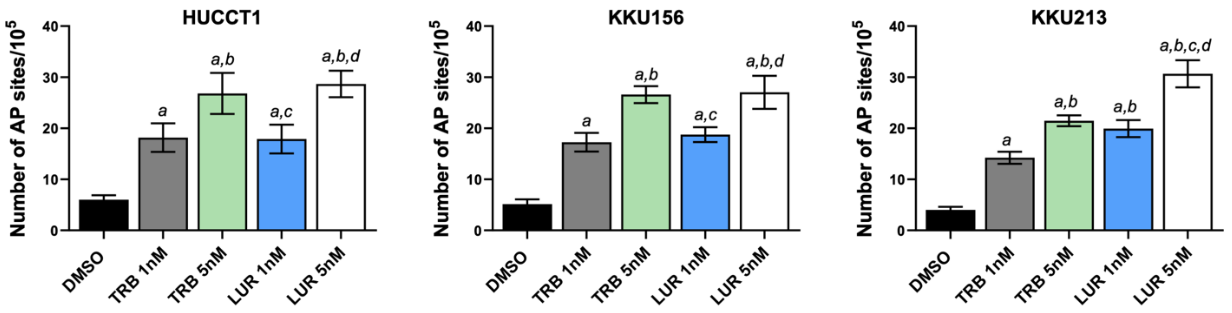

Supplementary Figure S5. Effects of trabectedin and lurbinectedin on the DNA damage of intrahepatic cholangiocarcinoma cell lines. The DNA damage assay, which assesses the formation of apurinic/apyrimidinic (AP) sites, one of the major types of DNA lesions, was applied on HUCCT1, KKU156, and KKU213 intrahepatic cholangiocarcinoma cell lines treated for 24 hours with trabectedin (TRB) and lurbinectedin (LUR) at 1 nM and 5 nM concentrations, respectively. Cells treated with solvent (DMSO) served as controls. Results are expressed as mean ± standard deviation of three independent experiments in triplicate. For statistical analysis, Tukey's multiple comparisons test was performed; at least $p < 0.001$; *a*, vs. DMSO; *b*, vs. 1 nM TRB; *c*, vs. 5 nM TRB; *d*, vs. 1 nM LUR.

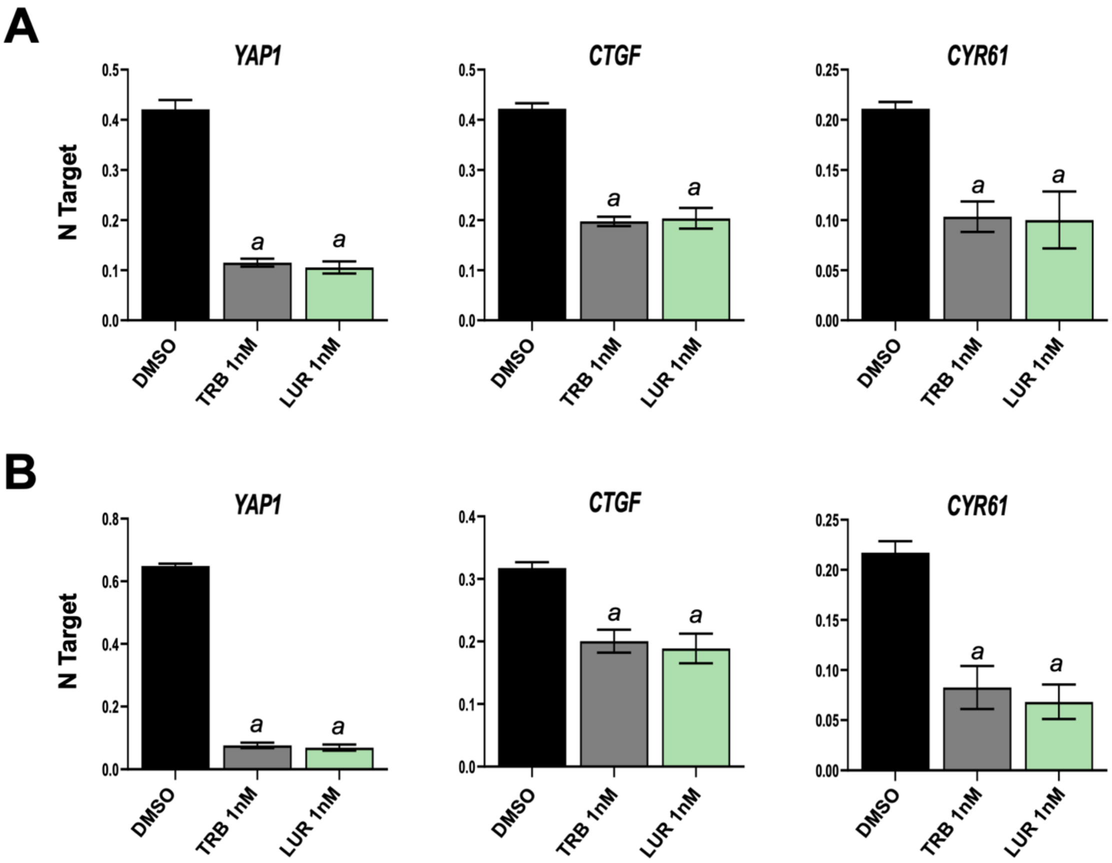

Supplementary Figure S6. Trabectedin and lurbinectedin induce the downregulation of the Hippo/YAP pathway in human intrahepatic cholangiocarcinoma cell lines. Quantitative real-time PCR was conducted in KKU055 (A) and HUCCT1 (B) intrahepatic cholangiocarcinoma cells subjected to trabectedin (TRB) and lurbinectedin (LUR) treatment at 1 nM concentration. mRNA levels of *YAP1, CTGF, and CYR61 w*ere determined. Quantitative values were calculated using the PE Biosystems Analysis software and expressed as N target (NT). NT = $2^{-\Delta Ct}$, where each sample's ΔCt value was calculated by subtracting the average Ct value of the target gene from the average Ct value of the *β-actin* gene. Results are expressed as mean ± standard deviation of three independent experiments in triplicate. For statistical analysis, Tukey's multiple comparisons test was performed; $p < 0.0001$; *a*, vs. DMSO.

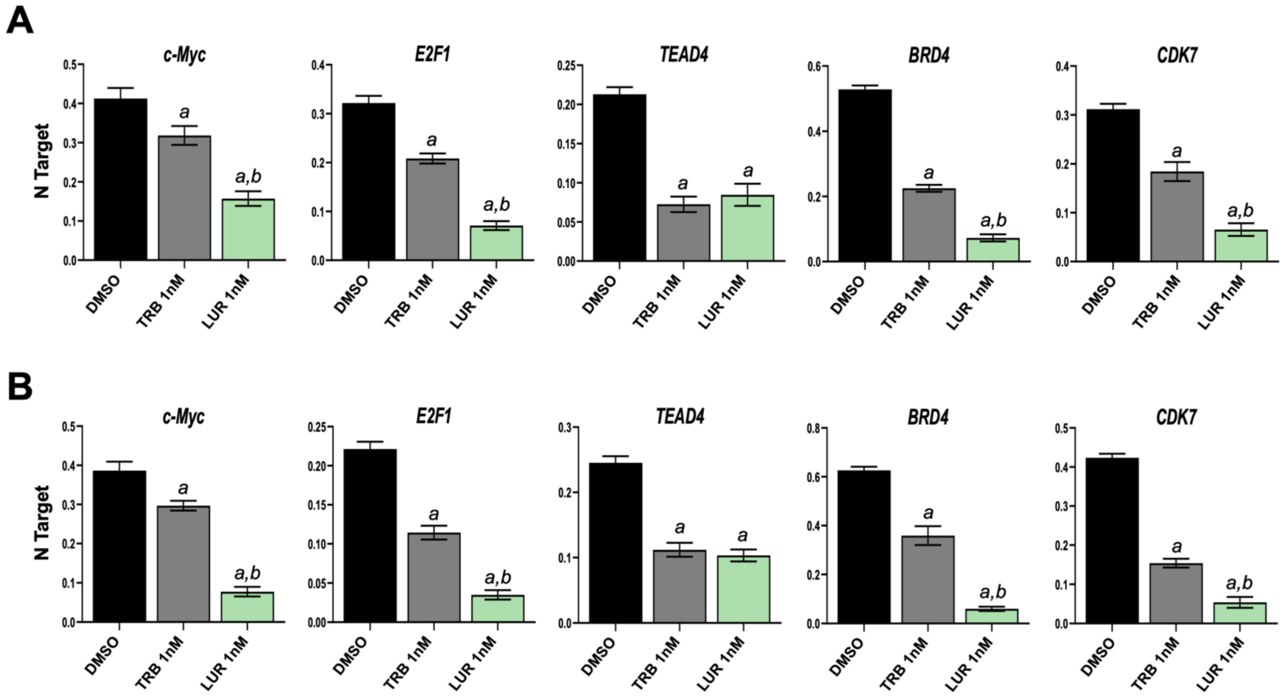

Supplementary Figure S7. Trabectedin and lurbinectedin induce the downregulation of oncogenic transcription factors in human intrahepatic cholangiocarcinoma cell lines. Quantitative real-time PCR was conducted in KKU055 (A) and HUCCT1 (B) intrahepatic cholangiocarcinoma cells subjected to trabectedin (TRB) and lurbinectedin (LUR) treatment at 1 nM concentration. mRNA levels of *c-Myc, TEAD4, E2F1, BRD4, and CDK7 w*ere determined. Quantitative values were calculated using the PE Biosystems Analysis software and expressed as N target (NT). NT = $2^{-\Delta Ct}$, where each sample's ΔCt value was calculated by subtracting the average Ct value of the target gene from the average Ct value of the *β-actin* gene. Results are expressed as mean ± standard deviation of three independent experiments in triplicate. For statistical analysis, Tukey's multiple comparisons test was performed; $p < 0.0001$; *a*, vs. DMSO; *b*, vs. 1 nM TRB.

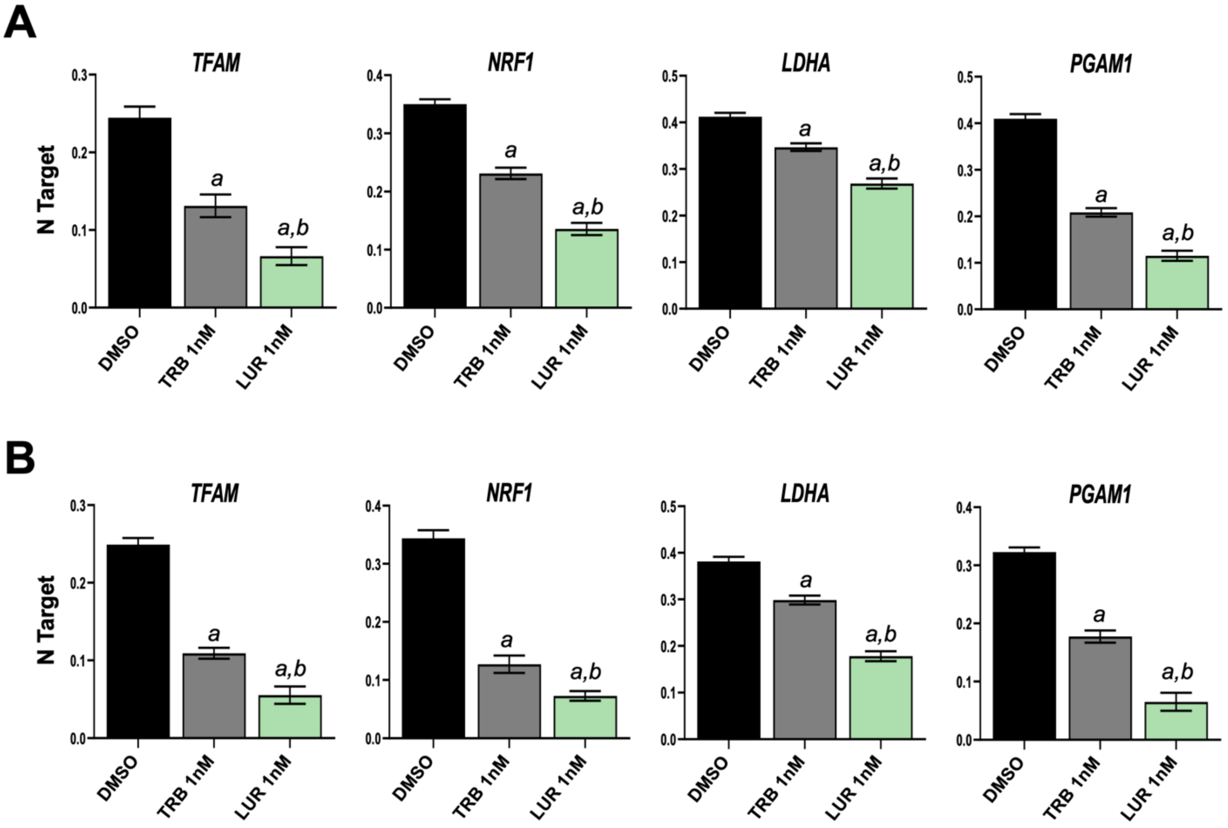

Supplementary Figure S8. Trabectedin and lurbinectedin induce the downregulation of c-Myc- and E2F1-regulated genes involved in mitochondrial biogenesis and glycolysis in human intrahepatic cholangiocarcinoma cell lines. Quantitative real-time PCR was conducted in KKU055 (A) and HUCCT1 (B) iCCA cells subjected to trabectedin (TRB) and lurbinectedin (LUR) treatment at 1 nM concentration. mRNA levels of *TFAM, NRF1, LDHA,* and *PGAM1* were determined. Quantitative values were calculated using the PE Biosystems Analysis software and expressed as N target (NT). $NT = 2^{-\Delta Ct}$, where each sample's ΔCt value was calculated by subtracting the average Ct value of the target gene from the average Ct value of the *β-actin* gene. Results are expressed as mean ± standard deviation of three independent experiments in triplicate. For statistical analysis, Tukey's multiple comparisons test was performed; $p < 0.0001$; *a*, vs. DMSO; *b*, vs. 1 nM TRB.
